# A Platform for Crowdsourced Foodborne Illness Surveillance: Description of Users and Reports

**DOI:** 10.2196/publichealth.7076

**Published:** 2017-07-05

**Authors:** Patrick Quade, Elaine Okanyene Nsoesie

**Affiliations:** ^1^ Iwaspoisoned.com New York, NY United States; ^2^ Institute for Health Metrics and Evaluation University of Washington Washington, WA United States

**Keywords:** foodborne illness surveillance, crowdsourced surveillance, foodborne diseases, infectious diseases, outbreaks, food poisoning, Internet, mobile, participatory surveillance, participatory epidemiology

## Abstract

**Background:**

Underreporting of foodborne illness makes foodborne disease burden estimation, timely outbreak detection, and evaluation of policies toward improving food safety challenging.

**Objective:**

The objective of this study was to present and evaluate Iwaspoisoned.com, an openly accessible Internet-based crowdsourcing platform that was launched in 2009 for the surveillance of foodborne illness. The goal of this system is to collect data that can be used to augment traditional approaches to foodborne disease surveillance.

**Methods:**

Individuals affected by a foodborne illness can use this system to report their symptoms and the suspected location (eg, restaurant, hotel, hospital) of infection. We present descriptive statistics of users and businesses and highlight three instances where reports of foodborne illness were submitted before the outbreaks were officially confirmed by the local departments of health.

**Results:**

More than 49,000 reports of suspected foodborne illness have been submitted on Iwaspoisoned.com since its inception by individuals from 89 countries and every state in the United States. Approximately 95.51% (42,139/44,119) of complaints implicated restaurants as the source of illness. Furthermore, an estimated 67.55% (3118/4616) of users who responded to a demographic survey were between the ages of 18 and 34, and 60.14% (2776/4616) of the respondents were female. The platform is also currently used by health departments in 90% (45/50) of states in the US to supplement existing programs on foodborne illness reporting.

**Conclusions:**

Crowdsourced disease surveillance through systems such as Iwaspoisoned.com uses the influence and familiarity of social media to create an infrastructure for easy reporting and surveillance of suspected foodborne illness events. If combined with traditional surveillance approaches, these systems have the potential to lessen the problem of foodborne illness underreporting and aid in early detection and monitoring of foodborne disease outbreaks.

## Introduction

The World Health Organization estimates that each year roughly 1 in 10 people worldwide experience illness after consuming contaminated food [1]. In the United States, an estimated 48 million people experience foodborne illness each year [2], resulting in costs of at least US $15.5 billion annually [3]. A majority of cases are unreported due to mild illness, limited public knowledge of reporting procedures, or absence of laboratory confirmation of disease [4]. Underreporting of foodborne illness makes foodborne disease burden estimation, timely outbreak detection, and evaluation of policies toward improving food safety challenging.

To augment traditional approaches (via phone, email, forms on department of health websites, fax, etc) to foodborne illness reporting, illness complaints submitted on social media and business review sites have been used by local departments of health for targeted restaurant inspections and foodborne disease outbreak surveillance [5-7]. Furthermore, foods implicated in foodborne illness reports on the business review site Yelp.com have also been shown to correlate with foods identified as the source of foodborne outbreaks by the US Centers for Disease Control and Prevention (CDC) [8]. These studies used passively collected information from social media and business review sites.

Alternatively, these data can be collected via crowdsourcing, which refers to the collection of information or completion of tasks by a large public audience [9]. Crowdsourcing or participatory surveillance has been used for public health monitoring of infectious diseases such as influenza and dengue [10,11]. These systems use mobile apps and the Internet to recruit and collect data on disease-specific symptoms. Examples include systems for monitoring influenza-like illness activity in Europe (eg, Influenzanet [12]) and the United States (eg, FluNearYou [13]); systems for identifying viruses causing acute respiratory infections at the community level (eg, GoViral [14]); and systems for reporting dengue and other mosquito-borne pathogens (eg, Kidenga [15]). Some of these systems have shown that participatory surveillance can enable timely ascertainment of susceptible and diagnosed cases of disease and has the potential to augment traditional disease surveillance systems [13,16].

Crowdsourced surveillance of foodborne illness can be useful in several ways. First, crowdsourced reporting of foodborne illness complaints is useful for general monitoring to enable targeted restaurant inspection [5] and identification of safety issues in the food production chain. Food can be contaminated at any point in the food production chain, and only a small proportion of foodborne illnesses are linked to outbreaks [17]. Early detection of food safety problems can prevent outbreaks. Second, information submitted through these platforms can aid in the early identification of unsafe food products, which can lead to product recall. Products can be unsafe due to contaminants or mislabeling that can result in illness or allergic reactions. Third, data submitted through these platforms can aid in detection and monitoring during foodborne disease outbreaks.

The objective of this paper was to present an efficient and easy-to-use data submission platform—Iwaspoisoned.com—for crowdsourced surveillance of foodborne illness.

## Methods

**Figure 1:**
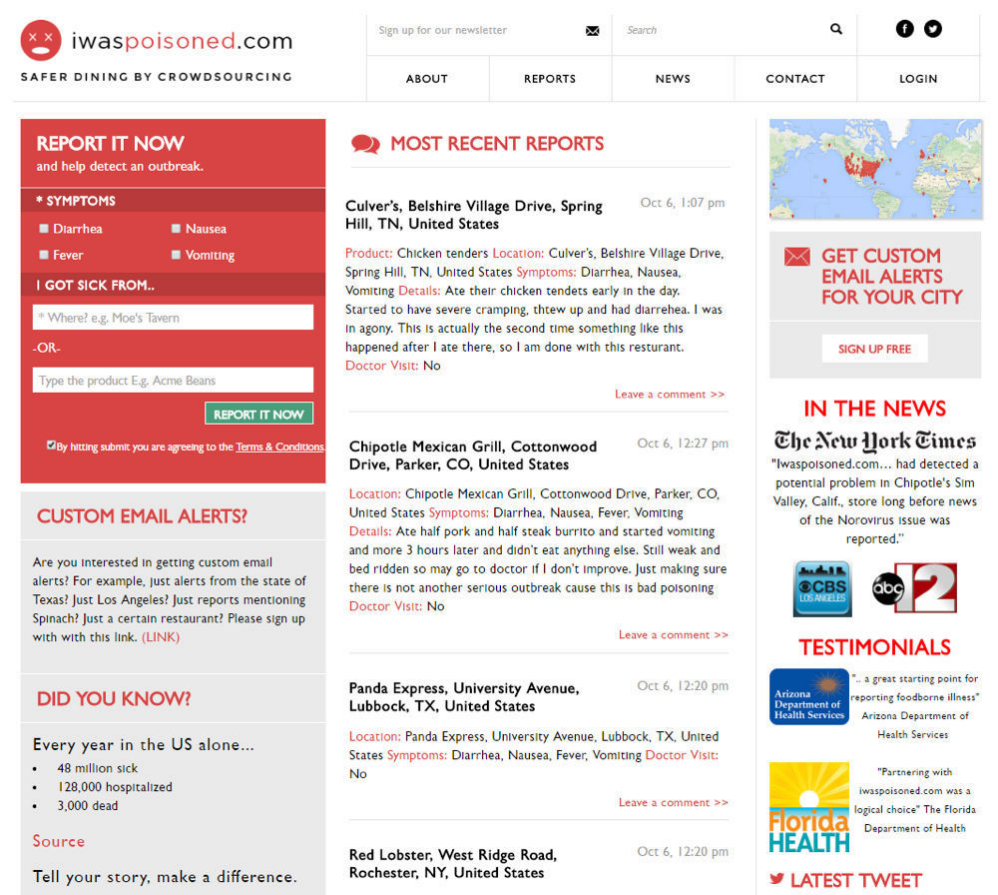
Screenshot of the Iwaspoisoned.com website.

Iwaspoisoned.com (Figure 1) is a freely accessible crowdsourcing system launched in 2009. Persons experiencing symptoms of a foodborne illness can submit information on the implicated foodservice business, foods consumed, date of visit, date of symptom onset, and number of affected individuals.

### Data Collection

A report can be submitted on Iwaspoisoned.com using the form displayed on the left in Figure 1. Upon clicking, “Report Now,” users will be directed to a second page where they are asked whether they saw a doctor and to provide additional details on their experience. Once the complaint (or report) is submitted, the unstructured data is formatted and stored in a database with each data element segregated into unique fields. The data fields include date, product details, restaurant details, description of experience, contact information (optional), and Internet Protocol (IP) address. Each report is assigned a unique identification number, and new reports default to the status of “Pending Review.”

To ensure data quality, human curators review reports for tone and accuracy of restaurant information. The restaurant details, which consist of the restaurant name and location, are validated using the Google search engine. If the restaurant name or location is missing or incorrect, the report is held for further review. We attempt to contact the user to obtain the missing details if the contact information is provided. In the event that we cannot validate the business location, the report is deemed invalid and is not published. Additionally, each description of a foodborne illness experience is carefully read to detect obscene or abusive language or language that suggests an attack on the business. Two examples of the 1700 submitted reports that have been excluded are presented.

Ate a piece of Trident Layers strawberry gum that my friend bought, felt a small (very tiny) trace of fever (mild headache, sore throat, mild loss of appetite) 1 hour later. Ilovehorseyrides.

Example 1

Location: Bikini Bottom I went to the Krusty Krab a few days ago. I ordered a Krabby Patty that shut was expensive n*gga. I got home and everything was all gucci. Next morning I saw dookie stains all over.my bed sheets and it smelled like ASS nigguh. Damn that sh*t was off chain. I suffered a Fever and I puked on my wifes lap and she bitch slapped me. F*ck Mr Krabs, Krusty Krab? More Like Krusty Crap. People, buy at the Chum Bucket, I hear their new human hot dog is a.must eat.

Example 2

Example 1 was excluded because of the inclusion of the nonsensical text—“ilovehorseyrides”—in this report and several other reports from the same IP address. All reports containing the nonsensical text were excluded. In Example 2, the restaurant cited was fictional, so the content was deemed inauthentic.

Furthermore, to eliminate spam, we verify IP addresses through IP lookup tools and identify suspicious patterns using a combination of automated and human review. We also consider a combination of timestamp, contact email, IP address, restaurant details, and description to identify multiple submissions of similar reports by the same user. If the duplicate reports are about the same incident and the details do not change, we assume the multiple submissions are because of user error. In such cases, only one report is published.

A report is only posted to the site and considered in review of larger trends after the authentication process. Food poisoning complaints posted on Iwaspoisoned.com are reviewed and compared with other reports to identify patterns in reported symptoms and geography. As foodborne diseases have varying incubation periods, there’s a possibility of individuals implicating the wrong foodservice business; therefore, surveillance is focused on identifying case clusters within a single report or across multiple reports rather than a single case. Some users submit reports while or right after experiencing illness, making it easier to identify and track disease clusters for timely outbreak control. Whenever a potential emerging or ongoing food safety issue is identified, the appropriate health department is contacted via email or phone and provided with details on the implicated business, symptoms, and contact information, if included in the report. The health department is contacted when two or more reports stating that multiple people experienced illness after eating at the same location are received within a span of 7 days.

### Survey

To better understand user demographics, we developed a survey that asked users about their gender (male/female) and age (under 18, 18-24, 25-34, 35-44, 45-55, 55+). The optional survey ran from February 6, 2016 to August 12, 2016, and was presented to every person who submitted a complaint. A second survey that focused on doctor visits and disease diagnosis was run from Feb 28, 2016 to July 10, 2016. The survey asked two questions: (1) Did you see a doctor? (Yes/No), and (2) If yes, was there a diagnosis? If there was a diagnosis, was the user diagnosed with a foodborne disease or another disease?

### Case Studies

In the last two years, the system has assisted in the detection and monitoring of outbreaks associated with at least three major food chains. Our knowledge on early detection involving communication with a health department is limited to outbreaks that were reported in the news. We present three examples: two local outbreaks and one of national attention because there were multiple outbreaks associated with the same chain across the United States. Note that these examples are meant to demonstrate the potential utility of this system and do not in any way suggest that foodborne outbreaks are limited to these restaurants.

## Results

### Data Collected

**Figure 2:**
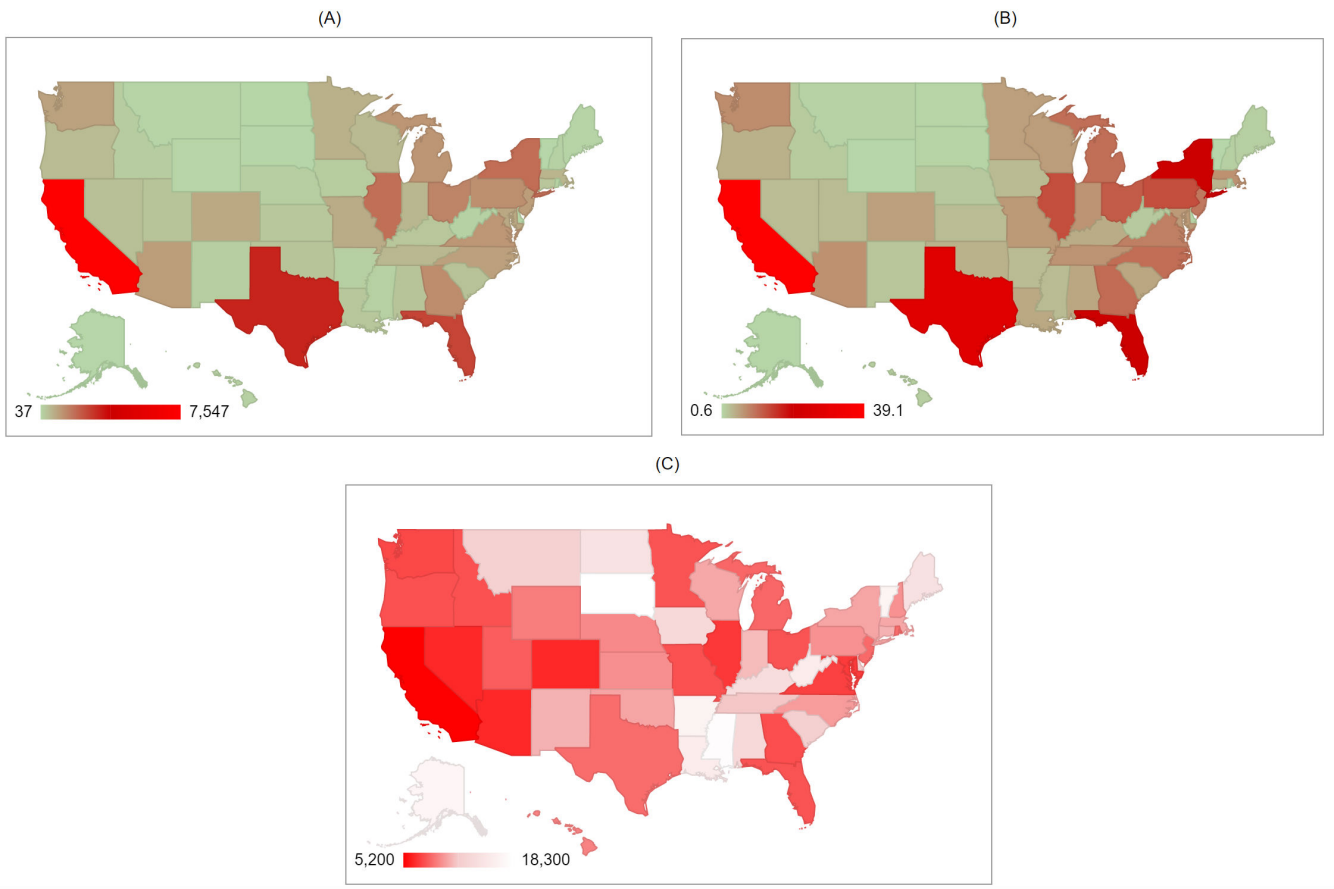
(A) heat map illustrating the distribution of foodborne reports by state with the legend from red to green, representing most to least. (B) heat map showing the most populous states in red, and the least populous in light green. (C) heat map showing per capita food poisoning reports per state. This is the population of the state divided by the number of reports received. The highest number of reports received per capita is in bright red at 1 for every 5200 people, the least number of per capita reports is in white at 1 for every 18,300 people.

As of October 6, 2016, 49,934 unique reports have been collected. Most reports originated from the United States (44,524/49,934, 89.17%), Canada (1070/49,934, 2.14%), United Kingdom (1050/49,934, 2.10%), and Australia (178/49,934, 0.36%). In the United States, the highest number of reports were received for California, accounting for 16.95% (7547/44,524) of all reports, followed by Texas with 7.10% (3161/44,524), Florida with 5.80% (2581/44,524), and New York with 4.24% (1886/44,524; Figure 2). In contrast, the lowest volume of reports was observed in Mississippi and South Dakota. Factors that could explain the low volume of reports in these states include low Internet access, low population density, and an absence of big cities.

Of the 44,119 users with precise information on the suspected business, 95.51% (42,139/44,119) and 2.63% (1159/44,119) attributed their illness to restaurants and grocery stores, respectively. The remainder were distributed across other business categories (Table 1). According to the most recent outbreak report from the US CDC for 2014, the most reported foodborne outbreaks associated with a single location were attributed to foods prepared in a restaurant: 485 (65%) outbreaks and 4780 (44%) associated illnesses [18].

### Survey Responses

The demographics survey had 4616 respondents. Approximately 67.5% (3118/4616) of respondents were under the age of 34, and 60.1% (2776/4616) of respondents were female (Table 2).

There were 7141 respondents to the doctor visit survey and 88.4% (6315/7141) did not see a doctor. Of those who saw a doctor, approximately 24.3% (201/826), 22.4% (185/826), and 5% (37/826) received a general diagnosis, no diagnosis, or other diagnosis (ie, not a pathogen associated with foodborne diseases), respectively. Approximately 7% (58/826) were either still awaiting results or did not want to share results. Of the remaining 41.8% (345/826), 32.8% (113/345) received a diagnosis of *Salmonella*, 27.5% (95/345) were diagnosed with *Norovirus*, 25.2% (87/345) with *Escherichia coli* (E coli), and 14.5% (50/345) with *Listeria*, *Campylobacter*, Colitis, Ciguatera toxin, Trichinosis, *Shigella*, *Clostridium*, and *Giardia lamblia*. According to the US CDC, *Norovirus* and *Salmonella* were the leading cause for confirmed foodborne disease outbreaks with a single etiology in the United States in 2014 [18].

**Table 1:** Distribution of reports across businesses.

Business	n (%)
Restaurant	42,139 (95.51)
Grocery/Supermarket/Warehouse	1159 (2.63)
Retail host	419 (1.0)
Convenience store/Gas station	130 (0.3)
Hotel/Resort/Casino	67 (0.2)
Packaged food product	57 (0.1)
Entertainment/Theme park/Festival	49 (0.1)
Airline	35 (0.1)
Airport	18 (0.0)
Shopping mall	11 (0.0)
University/School	11 (0.0)
Train station	6 (0.0)
Stadium	6 (0.0)
Hospital	5 (0.0)
Food truck	4 (0.0)
Community service/Church	2 (0.0)
Cruise	1 (0.0)
Total	44,119 (100.00)

**Table 2:** Survey of 4616 Iwaspoisoned.com users.

Age group in years	Female, n (%)	Male, n (%)	Total, n (%)
Under 18	281 (6.09)	158 (3.42)	439 (9.51)
18-24	772 (16.72)	519 (11.24)	1291 (27.97)
25-34	832 (18.02)	556 (12.05)	1388 (30.07)
35-44	453 (9.81)	304 (6.59)	757 (16.40)
45-55	251 (5.44)	164 (3.55)	415 (8.99)
55+	187 (4.05)	139 (3.01)	326 (7.06)
Total	2776 (60.14)	1840 (39.86)	4616 (100.00)

### Case Studies: Restaurant Outbreaks

We present three examples in which Iwaspoisoned.com detected clusters of similar illness linked to a restaurant before an official press release by the local health departments. Note, the case studies are meant to illustrate the potential of using this system to supplement state and local public health foodborne disease surveillance systems and in no way suggest that foodborne outbreaks are limited to these restaurants. See the US CDC and Surveillance Food Safety website [19] for more information on handling food and foodborne outbreaks.

First, in 2015, Chipotle, a major fast food company was identified as the source of several foodborne disease outbreaks across the United States. Between August and December of 2015, single- and multistate outbreaks were reported in California, Massachusetts, Minnesota, Wisconsin, Delaware, Illinois, Maryland, Kansas, New York, Oklahoma, Ohio, Oregon, and Washington.

On August 21, 2015, three reports citing a total of 7 people experiencing illness after consuming food at a Chipotle restaurant in Simi Valley, California, were submitted on Iwaspoisoned.com. On August 22, 6 new reports were submitted mentioning 23 sick individuals, and on August 23, 37 reports were submitted mentioning multiple sick persons; all implicating the same restaurant. Additional reports were submitted over the next several days, averaging approximately 5 to 10 per day. The persons mentioned in these additional reports experienced illness during the time frame of the outbreak; however, the individuals were either too sick to report in real time or discovered the website a few days post illness. The number of reports implicating Chipotle in Simi Valley increased from 1 to 30 within 2 days. On September 4, approximately 2 weeks after the initial report, the Ventura County Department of Health, California, confirmed a Norovirus outbreak linked to Chipotle.

**Figure 3:**
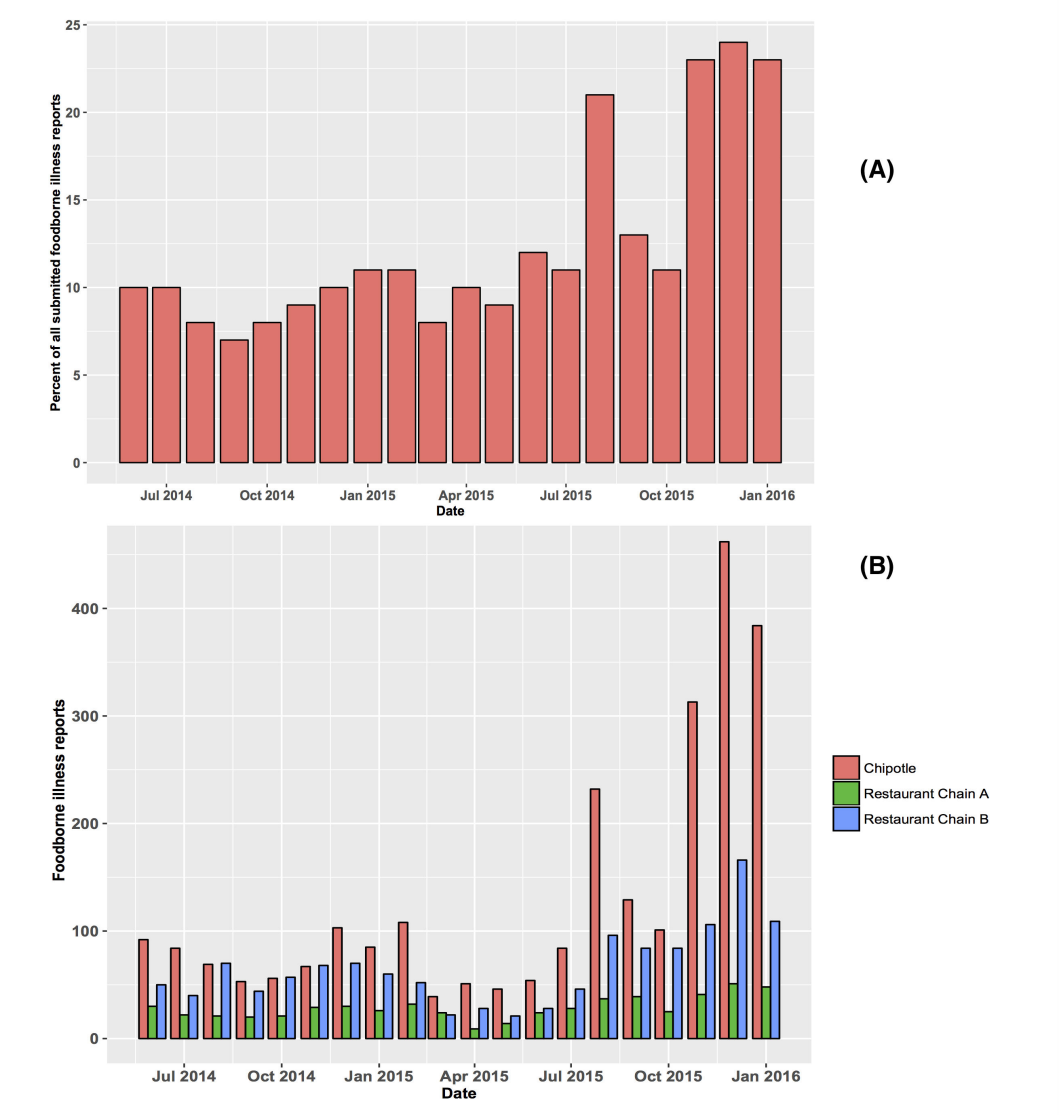
(A) The monthly percentage of suspected foodborne illness reports implicating Chipotle relative to all reports submitted to the system. Peaks in reporting are noted during the period when multiple outbreaks were reported. (B) Number of suspected foodborne illness reports implicating Chipotle, and Restaurant Chain A and Chain B, which have similar and greater than five times more store locations compared with Chipotle, respectively. The number of foodborne illness reports implicating Chipotle is significantly higher.

Data submitted to Iwaspoisoned.com suggested that as early as 2014, foodborne illness linked to Chipotle represented a significant proportion of reports submitted to the site (see Figure 3). The first peak in Figure 3 a is noted in August 2015 during the outbreak in California. Additional peaks in the foodborne illness reports implicating Chipotle were noted in November, December, and January. During this time, a multistate outbreak that began in October 2015 was reported in California, Illinois, Maryland, Minnesota, New York, Ohio, Oregon, Pennsylvania, and Washington. The CDC reports that 55 people were sickened with E coli O26 being the responsible pathogen; however, the contaminated food item was not identified [20]. In another outbreak that was reported in Boston in December 2015, Norovirus was implicated. Foodborne illness complaints implicating Chipotle were also higher during the outbreaks when compared with two other major food chains with similar or more locations across the United States (see Figure 3 b). Since then, Chipotle has taken several steps to improve sanitation at its facilities to prevent future outbreaks.

Second, on March 13, 2016, Iwaspoisoned.com received four reports citing 20 people becoming sick after eating at an Applebee’s in Corunna, Michigan, and alerted the Shiawassee County Health Department. On March 22, approximately a week after the initial report, the health department confirmed a Norovirus outbreak linked to that Applebee’s restaurant.

Third, in late December 2016, Iwaspoisoned.com contacted the Tacoma-Pierce County Health Department about a potential foodborne outbreak after receiving multiple foodborne illness reports associated with a Melting Pot franchise in Tacoma, Washington. Tacoma-Pierce County Health Department conducted an investigation and subsequently shut down the franchise for 24 hours.

## Discussion

The integration of social media and other digital data sources with traditional foodborne illness surveillance systems has the potential to improve foodborne illness surveillance in the United States. Data submitted on Iwaspoisoned.com can alert public health officers to clusters of foodborne illness reports as noted in the three case studies presented. Similar participatory surveillance systems developed for influenza-like illness have been shown to be useful in assessing influenza-like illness trends, risk factors, estimating attack rates, and care-seeking behavior [10-12,16,21,22].

### Limitations

Though crowdsourced surveillance has many advantages, there are some limitations to the use of these systems for public health surveillance. First, recruiting and maintaining participants is a major challenge. A study comparing different methods for recruiting participants suggested that offline enrollment is more effective than Internet-based campaigns [23]. However, Iwaspoisoned.com does not require enrollment and mainly requires responsiveness by the public. Potential approaches for increasing awareness include advertising and promotion, especially during disease outbreaks and collaboration with public health departments to increase the public’s understanding of the importance of reporting illness. Reports on Iwaspoisoned.com are accessed by state and local health departments in 90% (n=45) of US states to supplement existing programs on foodborne illness reporting. State food inspectors also use these data to generate alerts that focus on specific issues such as shellfish poisoning. Distributors can also monitor complaints associated with specific products or their foodservice business customers.

A second limitation is building a nationally representative sample. The demographics of users in the Iwaspoisoned.com system are skewed toward younger age groups; 67.5% are under the age of 34. We do not have data on the proportion of individuals who experience and report foodborne illness in each age category, therefore we cannot quantify representativeness. Younger and older persons who are at risk of more severe reactions to foodborne diseases might be underrepresented. Furthermore, studies suggest that representativeness in Internet-based systems and data sources for disease surveillance are influenced by factors such as gender, education, and income [24-26].

A third limitation involves the influence of news on disease reporting on social media and similar platforms. Though social media can aid in the early detection of disease outbreaks, it can also lead to over- and underreporting during outbreaks, which in some cases correlates with trends in news coverage during an outbreak [27,28]. Additionally, in the case of mild illness, individuals might be more likely to report suspected illness associated with an outbreak after public knowledge of the outbreak. Therefore, crowdsourced data might not always allow for early detection of outbreaks but can still enable monitoring of trends during outbreaks.

A fourth limitation is the validity of reports. Although there are several processes in place to assess the validity of foodborne illness complaints, these are not ensured to be completely effective. Therefore, Iwaspoisoned.com continues to refine the validation process and emphasize a focus on clusters of reports in the same locality or implicating the same business.

### Advantages

Despite these limitations, there are several advantages to using crowdsourcing for foodborne illness monitoring. Data from Iwaspoisoned.com suggests that the main pathogens reported by users with an official diagnosis of a foodborne disease are the same as those reported as major causes of foodborne illness in the United States. By enabling real-time reporting of illness, public health departments can encourage affected individuals to seek medical care and diagnosis.

Additionally, data from other platforms such as Yelp.com and Twitter.com can be integrated with data from Iwaspoisoned.com to track trends in reporting, identify changes in trends that are common across platforms, and correct for limitations in the individual sources. Furthermore, integrating data from these systems with traditional approaches to disease surveillance has the potential for mitigating the underreporting problem and aiding in timely reporting during outbreaks and disease burden estimation.

### Conclusions

Anyone can submit a report on Iwaspoisoned.com; however, the platform currently operates only in English. Future iterations would include additional languages. Additionally, the system will be updated to enable real-time geo-positioned location-based alerts for public health officers. Crowdsourced disease surveillance through systems such as Iwaspoisoned.com uses the ease and familiarity of social media to create an infrastructure for easy reporting and surveillance of foodborne illness events. Collaborations with local public health departments and the food service industry can improve data collection through such systems and create opportunities for educating the public regarding the importance of food safety and disease reporting.

## Abbreviations

CDC: Centers for Disease Control and Prevention IP: Internet Protocol
